# Low-temperature redetermination of tribenzyl­chloridotin(IV)

**DOI:** 10.1107/S160053680900289X

**Published:** 2009-01-28

**Authors:** Seik Weng Ng

**Affiliations:** a Department of Chemistry, University of Malaya, 50603 Kuala Lumpur, Malaysia

## Abstract

Compared to the previous studies [Ng (1997 ▶). *Acta Cryst.* C**53**, 56–58; Yin *et al.* (2005 ▶). *Huaxue Shiji*, **27**, 295–296], the redetermined structure of the title compound, [Sn(C_7_H_7_)_3_Cl], exhibits a doubled *c* unit-cell parameter. There are two mol­ecules in the asymmetric unit, with both Sn and both Cl atoms having 3 site symmetry. The Sn atoms have distorted SnClC_3_ tetra­hedral geometries and the mol­ecules inter­act by way of short Sn⋯Cl bridges [Sn⋯Cl = 3.418 (2) and 3.475 (2) Å], thereby forming chains propagating in *c*.

## Related literature

For the room-temperature structure of the title compound described in the *R*3 space group but with the unique *c* axis half as long, see: Ng (1997 ▶); Yin *et al.* (2005 ▶). For the direct synthesis of the title compound from metallic tin and benzyl chloride, see: Sisido *et al.* (1961 ▶).
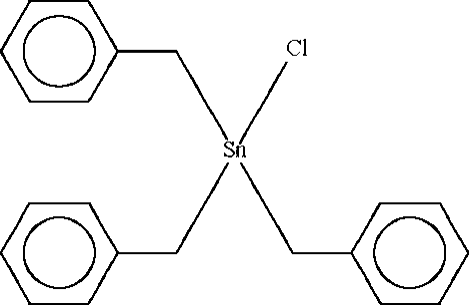


## Experimental

### 

#### Crystal data

[Sn(C_7_H_7_)_3_Cl]
*M* *_r_* = 427.52
Trigonal, 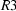

*a* = 16.7985 (2) Å
*c* = 11.6875 (2) Å
*V* = 2856.23 (6) Å^3^
*Z* = 6
Mo *K*α radiation
μ = 1.48 mm^−1^
*T* = 100 (2) K
0.40 × 0.08 × 0.06 mm

#### Data collection

Bruker SMART APEX diffractometer
Absorption correction: multi-scan (*SADABS*; Sheldrick, 1996 ▶) *T*_min_ = 0.589, *T*_max_ = 0.917
9077 measured reflections
2737 independent reflections
2431 reflections with *I* > 2σ(*I*)
*R*_int_ = 0.014

#### Refinement

*R*[*F* ^2^ > 2σ(*F* ^2^)] = 0.026
*wR*(*F* ^2^) = 0.074
*S* = 1.07
2737 reflections
139 parameters
1 restraint
H-atom parameters constrained
Δρ_max_ = 0.53 e Å^−3^
Δρ_min_ = −0.25 e Å^−3^
Absolute structure: Flack (1983 ▶), 1372 Friedel pairs
Flack parameter: −0.01 (4)

### 

Data collection: *APEX2* (Bruker, 2007 ▶); cell refinement: *SAINT* (Bruker, 2007 ▶); data reduction: *SAINT*; program(s) used to solve structure: *SHELXS97* (Sheldrick, 2008 ▶); program(s) used to refine structure: *SHELXL97* (Sheldrick, 2008 ▶); molecular graphics: *X-SEED* (Barbour, 2001 ▶); software used to prepare material for publication: *publCIF* (Westrip, 2009 ▶).

## Supplementary Material

Crystal structure: contains datablocks I, global. DOI: 10.1107/S160053680900289X/hb2901sup1.cif

Structure factors: contains datablocks I. DOI: 10.1107/S160053680900289X/hb2901Isup2.hkl

Additional supplementary materials:  crystallographic information; 3D view; checkCIF report

## Figures and Tables

**Table 1:** Selected bond lengths (Å)

Sn1—C1	2.146 (3)
Sn1—Cl1	2.392 (2)
Sn2—C8	2.143 (3)
Sn2—Cl2	2.403 (2)

**Fig. 1.**
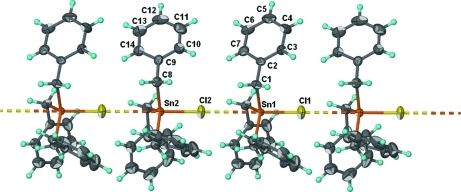
The molecular structure of (I); displacement ellipsoids are drawn at the 70% probability level, and H atoms as spheres of arbitrary radius. Only symmetry-independent atoms are labeled.

## supplementary crystallographic information

### Comment

The room-temperature structure of tribenzyltin(IV) chloride, (I), has been described in the *R*3 space group but with the unique *c*-axis half as long [a = 16.942 (1), c = 5.9187 (4) Å] (Ng, 1997; Yin *et al.*, 2005) as that found here.  Presumably, the two independent studies missed the weak reflections along the *c*-axis. In the present low-temperature study of (I) (Fig. 1), the *l* = 2*n* + 1 reflections are generally weak but are unambiguously present.  The crystal structure consists of [SnCI(C_7_H_7_)_3_] molecules (Tabl 1) linked axially by tin···chlorine bridges into a chain along the *c*-axis of the trigonal unit cell.

### Experimental

Tribenzyltin chloride was prepared from metallic tin and benzyl chloride in water (Sisido *et al.*, 1961) and was recrystallized from ethanol to yield colourless prisms of (I).

### Refinement

The H atoms were placed in calculated positions [C—H 0.95–0.99 Å, *U*_iso_(H) 1.2*U*_eq_(C)], and were included in the refinement in the riding-model approximation.

### Crystal data

**Table:**

[Sn(C_7_H_7_)_3_Cl]	*D*_x_ = 1.491 Mg m^−^^3^
*M**_r_* = 427.52	Mo *K*α radiation, λ = 0.71073 Å
Trigonal, *R*3	Cell parameters from 5172 reflections
Hall symbol: R 3	θ = 2.4–28.3°
*a* = 16.7985 (2) Å	µ = 1.48 mm^−^^1^
*c* = 11.6875 (2) Å	*T* = 100 K
*V* = 2856.23 (6) Å^3^	Prism, colorless
*Z* = 6	0.40 × 0.08 × 0.06 mm
*F*(000) = 1284	

### Data collection

**Table:**

Bruker SMART APEX diffractometer	2737 independent reflections
Radiation source: fine-focus sealed tube	2431 reflections with *I* > 2σ(*I*)
graphite	*R*_int_ = 0.014
ω scans	θ_max_ = 27.5°, θ_min_ = 2.2°
Absorption correction: multi-scan (*SADABS*; Sheldrick, 1996)	*h* = −21→21
*T*_min_ = 0.589, *T*_max_ = 0.917	*k* = −21→21
9077 measured reflections	*l* = −15→14

### Refinement

**Table:**

Refinement on *F*^2^	Secondary atom site location: difference Fourier map
Least-squares matrix: full	Hydrogen site location: inferred from neighbouring sites
*R*[*F*^2^ > 2σ(*F*^2^)] = 0.026	H-atom parameters constrained
*wR*(*F*^2^) = 0.074	*w* = 1/[σ^2^(*F*_o_^2^) + (0.0515*P*)^2^ + 0.375*P*] where *P* = (*F*_o_^2^ + 2*F*_c_^2^)/3
*S* = 1.07	(Δ/σ)_max_ = 0.001
2737 reflections	Δρ_max_ = 0.53 e Å^−^^3^
139 parameters	Δρ_min_ = −0.25 e Å^−^^3^
1 restraint	Absolute structure: Flack (1983), 1372 Fridel pairs
Primary atom site location: structure-invariant direct methods	Flack parameter: −0.01 (4)

### Fractional atomic coordinates and isotropic or equivalent isotropic displacement parameters (Å^2^)

**Table:**

	*x*	*y*	*z*	*U*_iso_*/*U*_eq_	
Sn1	0.3333	0.6667	0.500000 (15)	0.01587 (10)	
Sn2	0.3333	0.6667	1.00289 (2)	0.01945 (10)	
Cl1	0.3333	0.6667	0.29532 (14)	0.0388 (4)	
Cl2	0.3333	0.6667	0.79730 (13)	0.0364 (4)	
C1	0.4692 (2)	0.7751 (2)	0.5415 (3)	0.0231 (6)	
H1A	0.4805	0.7730	0.6242	0.028*	
H1B	0.5149	0.7659	0.4988	0.028*	
C2	0.4812 (2)	0.8672 (2)	0.5120 (3)	0.0214 (6)	
C3	0.5272 (2)	0.9129 (2)	0.4134 (3)	0.0353 (7)	
H3	0.5524	0.8858	0.3646	0.042*	
C4	0.5369 (3)	0.9971 (2)	0.3847 (3)	0.0475 (9)	
H4	0.5686	1.0272	0.3167	0.057*	
C5	0.5008 (3)	1.0378 (2)	0.4544 (4)	0.0438 (8)	
H5	0.5070	1.0954	0.4344	0.053*	
C6	0.4552 (2)	0.9929 (2)	0.5546 (4)	0.0361 (8)	
H6	0.4305	1.0204	0.6034	0.043*	
C7	0.4456 (2)	0.9089 (2)	0.5831 (3)	0.0283 (7)	
H7	0.4146	0.8791	0.6516	0.034*	
C8	0.4652 (2)	0.7841 (2)	1.0378 (3)	0.0269 (7)	
H8A	0.4828	0.7815	1.1180	0.032*	
H8B	0.5119	0.7831	0.9871	0.032*	
C9	0.4632 (2)	0.8710 (2)	1.0191 (3)	0.0259 (6)	
C10	0.4869 (2)	0.9179 (2)	0.9159 (3)	0.0417 (8)	
H10	0.5076	0.8957	0.8546	0.050*	
C11	0.4811 (3)	0.9968 (2)	0.9002 (4)	0.0531 (10)	
H11	0.4966	1.0271	0.8281	0.064*	
C12	0.4528 (2)	1.0317 (2)	0.9886 (4)	0.0482 (9)	
H12	0.4500	1.0863	0.9784	0.058*	
C13	0.4288 (2)	0.9860 (2)	1.0914 (4)	0.0401 (8)	
H13	0.4091	1.0091	1.1527	0.048*	
C14	0.4331 (2)	0.9067 (2)	1.1065 (3)	0.0311 (7)	
H14	0.4151	0.8756	1.1779	0.037*	

### Atomic displacement parameters (Å^2^)

**Table:**

	*U*^11^	*U*^22^	*U*^33^	*U*^12^	*U*^13^	*U*^23^
Sn1	0.01588 (11)	0.01588 (11)	0.01586 (17)	0.00794 (5)	0.000	0.000
Sn2	0.02122 (12)	0.02122 (12)	0.01592 (18)	0.01061 (6)	0.000	0.000
Cl1	0.0498 (6)	0.0498 (6)	0.0167 (5)	0.0249 (3)	0.000	0.000
Cl2	0.0470 (6)	0.0470 (6)	0.0152 (5)	0.0235 (3)	0.000	0.000
C1	0.0191 (14)	0.0214 (14)	0.0274 (14)	0.0092 (11)	−0.0005 (11)	0.0038 (11)
C2	0.0192 (13)	0.0178 (13)	0.0233 (13)	0.0062 (11)	−0.0037 (11)	0.0006 (10)
C3	0.0457 (19)	0.0255 (15)	0.0277 (14)	0.0125 (14)	0.0090 (14)	0.0008 (11)
C4	0.070 (2)	0.0274 (16)	0.0344 (19)	0.0163 (18)	0.0067 (16)	0.0085 (13)
C5	0.055 (2)	0.0231 (15)	0.050 (2)	0.0179 (17)	−0.0140 (17)	−0.0011 (14)
C6	0.0319 (18)	0.0280 (15)	0.048 (2)	0.0150 (14)	−0.0070 (14)	−0.0085 (14)
C7	0.0225 (14)	0.0270 (15)	0.0302 (16)	0.0086 (12)	−0.0012 (11)	−0.0059 (12)
C8	0.0231 (15)	0.0284 (16)	0.0276 (15)	0.0116 (13)	0.0011 (12)	−0.0012 (12)
C9	0.0246 (14)	0.0281 (15)	0.0208 (12)	0.0100 (12)	−0.0025 (11)	−0.0030 (11)
C10	0.047 (2)	0.0328 (17)	0.0280 (15)	0.0066 (16)	0.0013 (14)	0.0007 (13)
C11	0.063 (2)	0.0353 (19)	0.0365 (19)	0.0064 (18)	−0.0148 (17)	0.0104 (15)
C12	0.049 (2)	0.0255 (16)	0.063 (2)	0.0138 (17)	−0.0256 (18)	−0.0023 (16)
C13	0.0374 (18)	0.0338 (18)	0.050 (2)	0.0181 (15)	−0.0076 (15)	−0.0067 (15)
C14	0.0300 (16)	0.0278 (15)	0.0299 (16)	0.0103 (13)	0.0012 (12)	−0.0008 (12)

### Geometric parameters (Å, °)

**Table:**

Sn1—C1^i^	2.146 (3)	C5—C6	1.396 (5)
Sn1—C1	2.146 (3)	C5—H5	0.9500
Sn1—C1^ii^	2.146 (3)	C6—C7	1.379 (5)
Sn1—Cl1	2.392 (2)	C6—H6	0.9500
Sn1—Cl2	3.475 (2)	C7—H7	0.9500
Sn2—C8^i^	2.143 (3)	C8—C9	1.494 (5)
Sn2—C8	2.143 (3)	C8—H8A	0.9900
Sn2—C8^ii^	2.143 (3)	C8—H8B	0.9900
Sn2—Cl2	2.403 (2)	C9—C10	1.387 (4)
Sn2—Cl1^iii^	3.418 (2)	C9—C14	1.400 (4)
C1—C2	1.497 (4)	C10—C11	1.389 (5)
C1—H1A	0.9900	C10—H10	0.9500
C1—H1B	0.9900	C11—C12	1.383 (5)
C2—C3	1.387 (4)	C11—H11	0.9500
C2—C7	1.398 (4)	C12—C13	1.373 (5)
C3—C4	1.381 (4)	C12—H12	0.9500
C3—H3	0.9500	C13—C14	1.381 (5)
C4—C5	1.383 (5)	C13—H13	0.9500
C4—H4	0.9500	C14—H14	0.9500
			
C1^i^—Sn1—C1	115.06 (6)	C5—C4—H4	119.8
C1^i^—Sn1—C1^ii^	115.06 (7)	C4—C5—C6	119.0 (3)
C1—Sn1—C1^ii^	115.06 (6)	C4—C5—H5	120.5
C1^i^—Sn1—Cl1	103.05 (9)	C6—C5—H5	120.5
C1—Sn1—Cl1	103.05 (9)	C7—C6—C5	120.5 (3)
C1^ii^—Sn1—Cl1	103.05 (9)	C7—C6—H6	119.8
C1^i^—Sn1—Cl2	76.95 (9)	C5—C6—H6	119.8
C1—Sn1—Cl2	76.95 (9)	C6—C7—C2	120.6 (3)
C1^ii^—Sn1—Cl2	76.95 (9)	C6—C7—H7	119.7
Cl1—Sn1—Cl2	180.0	C2—C7—H7	119.7
C8^i^—Sn2—C8	116.46 (6)	C9—C8—Sn2	110.7 (2)
C8^i^—Sn2—C8^ii^	116.46 (6)	C9—C8—H8A	109.5
C8—Sn2—C8^ii^	116.46 (6)	Sn2—C8—H8A	109.5
C8^i^—Sn2—Cl2	100.98 (9)	C9—C8—H8B	109.5
C8—Sn2—Cl2	100.98 (9)	Sn2—C8—H8B	109.5
C8^ii^—Sn2—Cl2	100.98 (9)	H8A—C8—H8B	108.1
C8^i^—Sn2—Cl1^iii^	79.02 (9)	C10—C9—C14	117.0 (3)
C8—Sn2—Cl1^iii^	79.02 (9)	C10—C9—C8	122.9 (3)
C8^ii^—Sn2—Cl1^iii^	79.02 (9)	C14—C9—C8	120.1 (3)
Cl2—Sn2—Cl1^iii^	180.0	C9—C10—C11	121.4 (3)
Sn2—Cl2—Sn1	180.0	C9—C10—H10	119.3
C2—C1—Sn1	111.2 (2)	C11—C10—H10	119.3
C2—C1—H1A	109.4	C12—C11—C10	120.5 (3)
Sn1—C1—H1A	109.4	C12—C11—H11	119.7
C2—C1—H1B	109.4	C10—C11—H11	119.7
Sn1—C1—H1B	109.4	C13—C12—C11	118.9 (3)
H1A—C1—H1B	108.0	C13—C12—H12	120.5
C3—C2—C7	118.4 (3)	C11—C12—H12	120.5
C3—C2—C1	120.9 (3)	C12—C13—C14	120.6 (3)
C7—C2—C1	120.7 (3)	C12—C13—H13	119.7
C4—C3—C2	121.1 (3)	C14—C13—H13	119.7
C4—C3—H3	119.5	C13—C14—C9	121.6 (3)
C2—C3—H3	119.5	C13—C14—H14	119.2
C3—C4—C5	120.5 (3)	C9—C14—H14	119.2
C3—C4—H4	119.8		
			
C1^i^—Sn1—C1—C2	−41.4 (3)	C8^i^—Sn2—C8—C9	−31.7 (3)
C1^ii^—Sn1—C1—C2	−178.70 (16)	C8^ii^—Sn2—C8—C9	−175.18 (15)
Cl1—Sn1—C1—C2	69.9 (2)	Cl2—Sn2—C8—C9	76.6 (2)
Cl2—Sn1—C1—C2	−110.1 (2)	Cl1^iii^—Sn2—C8—C9	−103.4 (2)
Sn1—C1—C2—C3	−101.4 (3)	Sn2—C8—C9—C10	−92.7 (3)
Sn1—C1—C2—C7	78.1 (3)	Sn2—C8—C9—C14	84.8 (3)
C7—C2—C3—C4	−0.7 (5)	C14—C9—C10—C11	0.1 (5)
C1—C2—C3—C4	178.8 (3)	C8—C9—C10—C11	177.7 (3)
C2—C3—C4—C5	0.0 (5)	C9—C10—C11—C12	1.2 (5)
C3—C4—C5—C6	0.6 (6)	C10—C11—C12—C13	−1.3 (5)
C4—C5—C6—C7	−0.5 (5)	C11—C12—C13—C14	0.2 (5)
C5—C6—C7—C2	−0.2 (5)	C12—C13—C14—C9	1.1 (5)
C3—C2—C7—C6	0.8 (5)	C10—C9—C14—C13	−1.2 (5)
C1—C2—C7—C6	−178.7 (3)	C8—C9—C14—C13	−178.9 (3)

Symmetry codes: (i) −*y*+1, *x*−*y*+1, *z*; (ii) −*x*+*y*, −*x*+1, *z*; (iii) *x*, *y*, *z*+1.
